# Personal Economic Worries in Response to COVID-19 Pandemic: A Cross Sectional Study

**DOI:** 10.3389/fpsyg.2022.871209

**Published:** 2022-06-30

**Authors:** Imad Bou-Hamad, Reem Hoteit, Dunia Harajli, Dorota Reykowska

**Affiliations:** ^1^Suliman S. Olayan School of Business, American University of Beirut, Beirut, Lebanon; ^2^Clinical Research Institute, Faculty of Medicine, American University of Beirut, Beirut, Lebanon; ^3^Adnan Kassar School of Business, Lebanese American University, Beirut, Lebanon; ^4^NEUROHM Research Institute, Warsaw, Poland

**Keywords:** COVID-19, personal economic worries, individual differences, demographics, mental wellbeing factors

## Abstract

**Objectives:**

The emergence of the COVID-19 pandemic worsened Lebanon’s economic situation and generated worries about living conditions. This study aimed to explain personal economic worries patterns among Lebanese young adults while accounting for demographics and mental health characteristics.

**Methods:**

A total sample of 988 Lebanese responses were collected, using an online survey. The analysis was conducted using regression-based methods.

**Results:**

Men exhibited higher economic worries than women. Lower levels of economic worries among people with higher wages were more pronounced. Lebanese retirees experience the highest economic worries compared to other employment status groups. Individuals with higher life satisfaction are less concerned about the economy. Mental wellbeing factors were positively associated with personal economic worries.

**Conclusion:**

The current study presents a seminal insight into the differences in economic worries caused by COVID-19 pandemic among individuals in a developing country context.

## Introduction

The COVID-19 pandemic has had a devastating impact on global health care systems with a domino effect on every segment of human life (Nicola et al., 2020). On 30 January 2020, the World Health Organization (WHO) declared the outbreak of COVID-19 to be a global public health emergency putting countries with vulnerable health systems at high risk (Sohrabi et al., 2020). Till May 4, 2022, this pandemic has led to more than 510 million confirmed cases and over six million worldwide deaths. The emergence of COVID-19 caught the world off balance, creating the worst global health crisis of our time (Casale, 2020; Mallah et al., 2021). COVID-19 has profoundly impacted societies, businesses, and organizations around the world, thus impacting the global economy, where the negative effects of financial crises on long-term potential growth tend to be enduring (Nicola et al., 2020; Fuentes and Moder, 2021). A study conducted by Vitenu-Sackey and Barfi (2021) revealed that COVID-19 has inversely affected economic growth based on data collected from 170 countries (Vitenu-Sackey and Barfi, 2021). Additionally, over 80 countries have closed borders, restricted normal product and resource flows, ordered firms to close, encouraged individuals to self-quarantine, and closed schools, hampering industry and output (Barua, 2021; Hargreaves and Logie, 2020; Verma et al., 2021). According to Verma et al. (2021), the world’s top 10 economies are on the edge of collapse, including the United States, China, Japan, Germany, the United Kingdom, France, India, Italy, Brazil, and Canada (Verma et al., 2021). Consequently, many businesses have been forced to lay off employees in an attempt to survive and remain financially viable (Milani, 2020; Ansong and Turkson, 2022). Therefore, individuals are expected to experience increased economic worries during this pandemic particularly in countries with fragile financial and economic conditions. According to Roth et al. (2017), economic worries are linked to the concept of status anxiety (Roth et al., 2017).

Economic anxiety is a psychologically pernicious type of distress associated with detrimental consequences (Mann et al., 2020). A systematic review of the associations of mental health outcomes and economic recessions showed that periods of economic instability were substantially correlated with reduced mental wellbeing, elevated levels of common psychiatric illnesses, substance-related disorders, and suicidal behavior (Frasquilho et al., 2015). Fetzer et al. (2020) examined the underlying psychological processes that shape economic anxiety in a pandemic setting by evaluating the role of perceptions and knowledge about pandemic risk factors as well as the subjective mental models of infectious disease spread among individuals. Their findings indicate that heterogeneity in mental models of individuals influences crucially their understanding of the seriousness of a major global pandemic and affects their fears about the aggregate economy (Fetzer et al., 2020). Other research showed that the economic slump caused by extended periods of lockdown adversely impacted personal wellbeing, mental health and life satisfaction (de Pedraza et al., 2020; Ciardo et al., 2022). However, the variations in life satisfaction go beyond individual circumstances and are influenced by structural circumstances at the national level (inflation, unemployment, immigration) (Di Tella et al., 2001).

On March 18, 2020, the Lebanese government announced a state of general mobilization granting the authorities a legal mandate to enforce special measures against COVID-19, including border closures (airport, sea, and land) and public and private institutions (WorldBank, 2020). Even before a single death was announced, this strategy was regarded one of the harshest in the Middle East and North Africa (MENA) region (Kharroubi and Saleh, 2020). In Lebanon, the confirmed COVID-19 cases sharply increased by 72% between December 2020 and January 2021 with a weekly increase of more than 12,000 cases (World Health Organization [WHO], 2022. To combat the post-holiday COVID-19 rise, Lebanon implemented a strict new lockdown in January 2021, barring residents from even going grocery shopping and forcing them to rely on food deliveries (France24, 2021). Accordingly, a 24-h curfew has been imposed until January 25, 2021 as several hospitals began to run out of intensive care beds. This was followed by multiple lockdowns in 2021. Cases surged substantially again in December 2021, peaking at almost 57 000 weekly cases on January 31, 2022 (World Health Organization [WHO], 2022). According to the Lebanese Ministry of Public Health (MOPH), the overall number of affected individuals up to May 4, 2022 is more than 1.1 million, with over 10,000 deaths (Ministry of Public Health [MOPH], 2021).

Although the COVID-19 containment measures were relatively efficient at controlling the spread of the virus (Kharroubi and Saleh, 2020), they resulted in job losses and income reductions (World Food Programme [WFP], 2020) prompting businesses to close and depriving some people of work, especially informal day laborers. This was followed by a devaluation of the Lebanese lira vs. the US dollar and restrictions on international transfers out of the country (Devi, 2020; Mjaess et al., 2021). According to the World Bank, the economic and financial crisis in Lebanon is likely to be among the top 10, if not the top three, most catastrophic crises occurrences in the world since the mid-nineteenth century (WorldBank, 2021).

In parallel, the Syrian refugee crisis has cast a shadow over Lebanon’s economic situation since the past 8 years. The emergence of such a large refugee population in a small country facing economic recession, high unemployment, infrastructure constraints and environmental problems is increasingly threatening inter-community relationships and social stability (United Nations High Commissioner for Refugees [UNHCR], 2020). The total unemployment rate hit 25% in 2017 (Brihi et al., 2019) and this rate has risen to 30% in 2019 and 40% in 2020 (WorldBank, 2019; Karak et al., 2021). According to a recent report by the Food and Agriculture Organization of the United Nations, the unemployment rate has climbed to more than 40%, with more than 60% of the country’s young people unemployed (Food and Agriculture Organization [FAO], 2022).

These combined crises are expected to make the population of Lebanon particularly vulnerable to developing economic anxiety during the COVID-19 pandemic. To our knowledge, no studies assessing economic worries during the COVID-19 pandemic have been conducted in Lebanon. The main objective of this study is to explore personal economic worries and highlight the possible demographic and mental wellbeing factors moderating them.

## Materials and Methods

### Study Design and Participants

Our study was part of an international COVID-19-related research project comprising 20 nations initiated by a global research and technology institute. The project’s goal was to better understand people’s feelings, worries, behaviors, and opinions on the pandemic in multiple countries.

A cross-sectional data was collected using iCode smart test (Ohme et al., 2020; Bou-Hamad et al., 2021; Chkoniya et al., 2021) online-survey merging neuroscience, psychology, and artificial intelligence aspects. More specifically, this test demonstrates people’s reluctance to share their opinions and is a less complicated version of Implicit Association Testing (IAT), which is extensively used in psychology (Carpenter et al., 2019). The smart test records the speed and rhythm with which users touch the screen and assesses how they respond to questions using reaction time testing. It is expected that reported opinions with a short reaction time will mirror actual behavior (Fazio et al., 1989).

With the cooperation of eight specialists from the domains of psychology, sociology, marketing, and economics, the questionnaire was developed and piloted. Experts from the US, Poland, Singapore, Hong Kong, Portugal, and Switzerland collaborated on the project.

Between May 3 and May 31, 2020, a total of 988 Lebanese responses were collected. The survey was made available in three languages (Arabic, English, and French) reflecting the diversity of communication styles in the country. To preserve social distance and minimize virus propagation, the questionnaire was distributed online. As a result, convenience sampling was an appropriate method of data collection. Social media apps such as WhatsApp, Instagram, Facebook, and LinkedIn were all used to share a link to the survey along with a description. The link was also emailed to students, staff and faculty of the Lebanese American University (LAU), a prominent university in Lebanon. Prior to the start of the study, an approval was received from the Institutional Review Board at LAU.

Respondents were presented with a series of statements and their task was to answer if they agree with the sentence on the screen. Participants have been offered three possible responses: “YES,” “HARD to TELL,” and “NO.” The explicit and implicit responses were simultaneously recorded, where implicit responses are represented by the speed of reaction. To make sure that the measured response time (RT) is not biased by the position of the mouse, a control screen (Advanced Button) was introduced. It was presented before each statement and forced the mouse position that kept the same distance to both yes and no answers. It is of note that RT is measured in milliseconds (ms).

The test part was preceded with a calibration phase, that consisted of three parts: (i) *Familiarization with the scale.* The aim of this part was to acquaint respondents with the answer scale and the position of the buttons on the screen; (ii) *Familiarization with the purpose of the task and motoric warm up.* A series of statements were presented explaining the purpose of the task. Participants were informed that the test measures opinions and certainty. Twelve such screens were presented introducing participants to the task in the main part of the test; (iii) *Increasing participants engagement in the task.* To make sure that respondents pay attention to the presented statements a validation box showed up twice, asking to indicate what statement was shown on the previous screen. If the response was wrong a message showed up asking to work more carefully (“Please work carefully”).

### Measures

Expressing attitudes and describing behavior around sensitive topics, full of emotional load are very prone to distortions. Political correctness, post rationalizations or auto presentation needs are all important aspects that frequently influence explicit, declarative answers. Self-reported measures, according to Greenwald and Banaji (1995), are insufficient to capture implicit processes, necessitating the use of indirect techniques to improve the accuracy of explicit responses (Greenwald and Banaji, 1995). The majority of these tools employ the response time or reaction time of respondents as a proxy for implicit processes (Ohme et al., 2020).

The approach of Fazio’s theory of attitude accessibility is followed in this study’s RT measurement (Fazio and Williams, 1986). Attitudes that are stronger, are more accessible and therefore expressed with a shorter response time (Fazio and Williams, 1986). In fact, compared to traditional questionnaires, response time measurement provides data that are less influenced by self-presentation bias or social desirability (Greenwald et al., 2009).

In addition to demographics (gender, income, age, education, living status, number of children, employment status, and city size), the surveys included 56 individual statements. These statements mainly revolved around six areas: perceptions, attitudes, emotions, knowledge, politics, and economy. In the current study, we measured the personal economic worries construct and the three-mental wellbeing constructs, with a total of 16 statements reported in Supplementary Table 1. The considered mental wellbeing factors: *life satisfaction*, *health worries*, and *social wellbeing concerns* are believed to be interrelated with economic worries.

In this research, the construct items used to measure health worries, life satisfaction, and social wellbeing concerns are obtained from the literature and consistent with a recent study conducted by Bou-Hamad et al. (2021). Additionally, economic worry statements are derived from COSMO single-item measures (Betsch et al., 2020). Responses to the constructs item were converted to a 4-Likert scale (Bou-Hamad et al., 2021). We measured the internal consistency of the construct items using Cronbach’s alpha, the most common measure of reliability. Cronbach’s alpha values were nearly between 0.6 and 0.7. A value between 0.6 and 0.7 suggests an acceptable level of reliability (Ursachi et al., 2015). Subsequently, we created the personal economic worries (PEW) variable and the three-mental wellbeing factors as average response scores.

## Results

### Descriptive Statistics

Table 1 summarizes the demographic characteristics of the participants considered for this study. Our sample included participants from different age groups, education levels, and income classes. The median income class was between 1,000,000 LBP and 2,500,000 LBP. Almost two-thirds of the participants were women, with the majority (60%) between 18 and 25 years of age. The majority of participants (90%) earned a bachelor’s or higher degree or continue to study at university, with one third living in large cities (more than 100,000 inhabitants). Couples with children constituted 23% of the sample. In terms of employment status, 34% were working, 48% were students, and 12% were unemployed.

**TABLE 1:** Descriptive statistics.

Variable	n (%)
**Age (years)**	
18–25	589 (59.6)
26–35	197 (19.9)
36–49	133 (13.5)
50–64	46 (4.7)
≥65	9 (0.9)
Missing	14 (1.4)
**Gender**	
Men	352 (36.6)
Women	609 (63.4)
Missing	27 (2.7)
**Education level**	
Elementary school	8 (0.8)
Brevet degree or 9th grade	9 (0.9)
BACC II or High school	60 (6.1)
Vocational	33 (3.3)
Bachelor’s degree or higher	878 (88.9)
**Living status**	
Living alone	90 (9.1)
Living alone with children	57 (5.8)
Couple (married, or not) without children	42 (4.3)
Couple with children	226 (22.9)
Living with parents	573 (58.0)
**Children**	
No	720 (72.9)
≥1	268 (27.1)
**City size (people)**	
<10,000	291 (29.5)
Between 10,000 and 14,999	158 (16.0)
Between 15,000 and 99,999	67 (6.8)
Between 20,000 and 49,999	72 (7.3)
Between 50,000 and 99,999	71 (7.2)
≥100,000	329 (33.3)
**Employment status**	
Employed	339 (34.3)
Student	469 (47.5)
Entrepreneur	48 (4.9)
Retired	9 (0.9)
Homemaker	3 (0.3)
Unemployed	120 (12.1)
**Monthly income (LBP^‡^)**	
≤600,000	108 (19.0)
Between 600,000 L.L and 1,000,000	101 (17.8)
Between 1,000,000 and 2,500,000	171 (30.2)
Between 2,500,000 and 5,500,000	94 (16.6)
Between 5,500,000 and 8,500,000	59 (10.4)
≥8,500,000	34 (6.0)
Missing	421 (42.6)

*^‡^LBP is Lebanese pound (1 USD = 12,800 LBP).*

### Demographic Differences

We used ordinary least squares (OLS) regression to study the demographic differences in terms of economic worries. However, the constant-variance assumption (homoscedasticity) was not met. Therefore, robust standard errors method is employed to correct heteroscedasticity. The analysis is performed with R language (Version 3.4.4) and the results are reported in Table 2.

**TABLE 2:** Regression analysis to explain relationships between personal economic worries and demographics.

Variable	β (SE)	*p*
**Age**	0.02 (0.04)	0.695
**Gender^†^**	0.17 (0.05)	<**0.001**
**Education**	0.01 (0.04)	0.852
**Children**	0.06 (0.08)	0.505
**City size**	−0.01(0.01)	0.421
**Monthly income**	−0.10(0.02)	<**0.001**
**Employment status^§^**		
Employed	0.10 (0.10)	0.336
Student	0.00 (0.11)	0.999
Entrepreneur	0.04 (0.16)	0.813
Retired	0.33 (0.14)	**0.022**
**Living status^¥^**		
Living alone with children	−0.09(0.14)	0.491
Couple (married, or not) without children	0.15 (0.11)	0.201
Couple with children	−0.04(0.11)	0.706
Living with parents	−0.02(0.08)	0.779

*β Beta(s) are regression coefficients; SE standard errors are shown in parentheses, p is the p-value.*

*^†^Woman was used as a reference group for gender.*

*^§^Unemployed was used as a reference group for employment status.*

*^¥^Living alone was used as a reference for living status.*

*Bold values represent significant p-values < 0.05.*

There is a significant association between gender and economic worries (*p* < 0.001). The positive coefficient (β = 0.17) indicates that men are more anxious than women (the reference group) about the economic conditions affected by COVID-19. People with higher income exhibited lower level of economic worries (β = 0.17, *p* < 0.001). Employment status is statistically significant for retirees (*p* < 0.05). Average economic worry was higher for retirees (β = 0.33) than other groups of the employment status. Economic worry was not significantly associated with age, education, number of children, size of the city, and living status.

### Differences Between Men and Women

Economic worries was significantly different among men and women. Therefore, we run further regression analysis to highlight possible differences in economic worries between genders across the demographic characteristics (Table 3).

**TABLE 3:** Differences in economic worries between men and women across demographics.

	Women		Men	
Variable	β w (SE)	*p*	β M (SE)	*p*
**Age**	0.05 (0.07)	0.477	−0.02(0.06)	0.798
**Education**	−0.02(0.00)	0.720	0.08 (0.05)	0.075
**Children**	0.12 (0.11)	0.310	−0.01(0.13)	0.962
**City size**	0.00 (0.01)	0.998	−0.03(0.01)	**0.045**
**Monthly income**	−0.11(0.02)	**<0.001**	−0.09(0.03)	**0.002**
**Employment status^§^**				
Employed	0.13 (0.12)	0.288	0.10 (0.14)	0.502
Student	−0.01(0.14)	0.956	0.07 (0.15)	0.623
Entrepreneur	0.08 (0.19)	0.687	−0.15(0.22)	0.492
Retired	−	-	0.56 (0.18)	**0.002**
**Living status^¥^**				
Living alone with children	−0.18(0.18)	0.341	0.08 (0.19)	0.685
Couple (married, or not) without children	0.02 (0.13)	0.905	0.44 (0.20)	**0.029**
Couple with children	−0.23(0.12)	**0.049**	0.26 (0.13)	**0.046**
Living with parents	−0.09(0.1)	0.402	0.09 (0.11)	0.435

*β Beta(s) are regression coefficients; SE standard errors are shown in parentheses; p is the p-value.*

*^§^Unemployed was used as a reference group for employment status.*

*^¥^Living alone was used as a reference for living status.*

*Bold values represent significant p-values < 0.05.*

Both women and men with higher income perceived lower economic worries. However, women were slightly less worried than men (β_*w*_ = −0.11; β_*M*_ = −0.09). The size of city was associated with economic worries among men, but not among women (β_*M*_ = −0.3; *p* < 0.05). All retirees were men and had high economic worries. In couples without children, men experienced economic worries (β_*M*_ = 0.44; *p* = 0.029) while women did not. As for couples with children, women perceived lower economic worries than men (β_*w*_ = −0.23; β_*M*_ = 0.26). While the level of education was not a significant predictor of women’s economic worries (*p* > 0.05), it was borderline significant for men (*p* = 0.075). No differences between men and women in economic worries have been observed across age and the number of children.

### Associations Between Mental Wellbeing Factors and Economic Worries

We first conducted a correlational analysis to examine the associations of the mental wellbeing constructs and personal economic worries. Health worries were positively correlated with economic worries (*r* = 0.47; *p* < 0.001). Similarly, there was a positive correlation between economic worries and wellbeing concerns (*r* = 0.41; *p* < 0.001). Unlike health worries and social wellbeing concerns, life satisfaction shows a negative association with economic worries (*r* = −0.15; *p* < 0.001).

Controlling for demographics, we estimated a regression model for economic worries that included the mental wellbeing constructs. The results reported in Table 4 show that the direction of influence of each construct along with its statistical significance have not changed.

**TABLE 4:** Regression analysis to explain relationships between personal economic worries and mental wellbeing factors accounting for demographics.

Variables	β (SE)	*P*-value
**Age**	0.03 (0.04)	0.495
**Gender**	0.10 (0.05)	**0.047**
**Education**	0.02 (0.07)	0.759
**Children**	−0.004(0.10)	0.961
**City size**	−0.02(0.01)	0.217
**Monthly income**	−0.09(0.02)	<**0.001**
**Employment status** ^§^		
Employed	0.10 (0.10)	0.288
Student	−0.01(0.10)	0.908
Entrepreneur	0.03 (0.15)	0.801
Retired	0.11 (0.14)	0.433
**Living status^¥^**		
Living alone with children	−0.16(0.15)	0.284
Couple (married, or not) without children	−0.10(0.16)	0.522
Couple with children	−0.02(0.12)	0.856
Living with parents	−0.05(0.09)	0.546
**Health worries**	0.20 (0.05)	**<0.001**
**Life satisfaction**	−0.08(0.04)	**0.047**
**Social wellbeing concerns**	0.22 (0.05)	**<0.001**

*β Beta(s) are regression coefficients; SE standard errors are shown in parentheses; p is the p-value.*

*^§^Unemployed was used as a reference group for employment status.*

*^¥^Living alone was used as a reference for living status.*

*Bold values represent significant p-values < 0.05.*

## Discussion

The Lebanese economy experienced a sharp and sustained recession toward the end of 2019. The emergence of the COVID-19 pandemic aggravated the economic situation and raised fears in the society (WorldBank, 2021). This study attempted to assess personal economic worries accounting for demographic and mental wellbeing related factors. While some studies do not show a significant difference between genders with regards to level of personal economic anxiety and financial hardships (Fetzer et al., 2020; Mann et al., 2020), our findings show a significant association between economic worries and gender. Research revealed that women worry more than men and are affected by the stress of a receding economy, we found that men expressed higher levels of worries (Bareket-Bojmel et al., 2020; Di Crosta et al., 2020). This outcome could be explained by the heavily patriarchal nature of Lebanese society where men are usually the household’s principal financial providers (Tlaiss and Kauser, 2011). Accordingly, with the severe COVID-19 pandemic shutting down businesses and work, it is natural for men to feel more threatened and worried about losing their job or not work enough hours to cater to their households’ basic needs.

At the income level, higher wages translated into lower levels of economic worries. This result concurs with previous studies in the literature (Lei et al., 2020; Mann et al., 2020). Nonetheless, contrary to Mann et al. (2020), our study found that retirees were experiencing the highest economic worries compared to other employment status groups. This finding is not surprising given that Lebanon lacks the safety net associated with welfare state programs such as old age security. In addition to this structural problem, current pensioners who traditionally would rely on their lifelong savings, have not been able to access them and seen them drastically devalued since late 2019.

Recent studies have shown that age and number of children are significantly related to economic anxiety (Lei et al., 2020; Mann et al., 2020). Younger people fear the economic situation and the higher risk of unemployment (Fetzer et al., 2020; Mann et al., 2020), and households with children exhibit higher economic anxiety (Mann et al., 2020). While these associations have been established in the literature, they have not been detected in our study.

Moving forward, we highlighted economic worries differences among men and women accounting for demographics. The direction of influence of income on economic worries was the same for both genders, where those earning higher salaries worry less about their economic conditions. In contrast, men show higher economic worries among couples with children. This can be justified, given men are primarily responsible for providing financial assistance to their family in Lebanon. Additionally, a study conducted by Rosman et al. (2021) showed that men were more concerned with economic and social consequences (Rosman et al., 2021).

Furthermore, the size of the city was significantly associated with economic worries in men but not in women. Men who live in bigger cities have less economic worries than those living in smaller towns. This finding can be explained by the fact that employment opportunities in larger cities are higher. On the other hand, we expected the number of children to be a candidate predictor of personal economic worries because more children can increase a household’s financial burden and hence cause parents to be more concerned about their financial condition, but this was not the case. However, as long as personal economic worries are regarded a mental health outcome, this conclusion does not contradict the literature. According to Dai et al. (2020), the number of children is unrelated to any of the mental health outcomes studied in their research, including anxiety, depression, and distress (Dai et al., 2020).

In addition to demographics, we explored the associations between personal economic worries and mental wellbeing factors. We found that people who are more satisfied with their lives tend to be less worried about the economy. This is consistent with previous literature on life satisfaction and economic worries, where researchers have concluded that life satisfaction declines as worries increases during the pandemic (Roth et al., 2017).

A significant positive association between health worries and economic worries was also discovered in our research. This is similar to the findings of Bareket-Bojmel et al. (2020), who concluded that the intensity of health anxiety is comparable to that of economic anxiety. Moreover, we found that persons who are concerned about their social wellbeing also had higher levels of economic worries.

## Limitations

Like any research, this study has some limitations. Participants have been recruited conveniently through social media platforms. While convenience sampling might not guarantee the generalizability of findings, it can still be considered a valid procedure for establishing the likelihood of possible associations between variables. Yet, online surveys fail to include illiterates and economically disadvantaged groups who are unable to purchase computers or smart phones. Online surveys, on the other hand, remain useful data collection tools during the pandemic to conduct research while minimizing contagion risk. Another limitation was the low representation of certain groups, such as retirees. The related findings are therefore to be treated with caution. It is worth noting that, almost 80% of young adults (18–35 years) were included in this study. This age group makes up a significant proportion of the entire Lebanese population (32%) and also make up 98% of active internet users in the Mena region (Statista, 2017). As a result, we may safely conclude that our sample is typical of the population of internet-literate young adults in Lebanon, and that our findings apply to them. Additionally, in future studies, we will assess both state and trait anxiety because similar individual’s characteristics may also contribute to economic concerns.

COVID-19 unexpectedly appeared in our lives, therefore understanding its long-term impact on people’s perceptions needs more research. The current study presents a seminal insight into the differences in economic worries caused by COVID-19 pandemic among individuals in a developing country context. Given the identified limitations, notably the use of a convenience sample, the findings of the current study should be taken as suggestive rather than conclusive.

## Conclusion

The purpose of this study was to explore people’s personal worries in Lebanon in response to the COVID-19 pandemic, as well as to highlight differences in demographics and mental wellbeing characteristics. Findings among internet-literate Lebanese young adults revealed that men reported higher levels of economic worries than women, whereas those with higher salaries had lower levels of economic worries. Furthermore, health worries and mental wellbeing concerns were positively associated with personal economic worries. The findings provide a roadmap for decision makers and global health practitioners to craft interventions that mitigate personal economic concerns associated with COVID-19 crisis and other related mental wellbeing issues.

## Data Availability Statement

The datasets presented in this study can be found in online repositories. The names of the repository/repositories and accession number(s) can be found below: We made the data publicly available by depositing it in a data bank maintained by The American University of Beirut’s Libraries. The datasets used and/or analyzed during the current study are available from the corresponding author on reasonable request or *via* the following: http://hdl.handle.net/10938/22923.

## Ethics Statement

An approval was received from the Lebanese American University Institutional Review Board. Data collection was performed in accordance with the Declaration of Helsinki. All appropriate measures were taken to ensure participants’ anonymity and information confidentiality. Prior to filling the survey, all participants provided informed consent online.

## Author Contributions

IB-H created the conceptual framework, analyzed, and interpreted the data. RH performed the data curation and conducted the literature review. IB-H and RH were major contributors in writing the manuscript. DH collected the data and contributed to the discussion. DR contributed to the methodology of data collection. All authors read and approved the final manuscript.

## Conflict of Interest

The authors declare that the research was conducted in the absence of any commercial or financial relationships that could be construed as a potential conflict of interest.

## Publisher’s Note

All claims expressed in this article are solely those of the authors and do not necessarily represent those of their affiliated organizations, or those of the publisher, the editors and the reviewers. Any product that may be evaluated in this article, or claim that may be made by its manufacturer, is not guaranteed or endorsed by the publisher.
