# Supplementary material for: Personal Economic Worries in Response to COVID-19 Pandemic: A Cross Sectional Study
Source: Front Psychol. 2022 Jun 30;13:871209. doi: 10.3389/fpsyg.2022.871209 (PMC9280425; doi:10.3389/fpsyg.2022.871209)
Supplement: Supplementary file 1 [file Table_1.DOCX]

**Table A1** Items forming the considered constructs in the study

| Construct | Questions |
| --- | --- |
| **Personal economic worries (PEW)** | I'm worried that there will not be enough of basic products in the stores |
|  | I'm worried about my financial situation |
|  | I'm worried I will lose my job |
|  | I'm worried that our country will run out of money |
|  | I believe that stocking up on essential items is necessary |
| **Health worries** | I'm afraid of becoming infected with coronavirus |
|  | I'm worried about my own health |
|  | I'm worried about the health of my children |
|  | I'm worried about the health of my older family members |
| **Life satisfaction** | I'm satisfied with my country  I'm satisfied with my family  I'm satisfied with myself |
| **Social well-being concerns** | I'm worried about not being able to meet with friends |
|  | I'm worried about not being able to meet with my family |
|  | I'm worried that living in isolation will negatively affect my well-being |
|  | I'm afraid that life in isolation will negatively impact my health |
